# Antioxidant Ready-to-Use Grape Pomace Extracts Recovered with Natural Eutectic Mixtures for Formulation of Color-Rich Gummies

**DOI:** 10.3390/foods13172840

**Published:** 2024-09-07

**Authors:** Julia Trentin, Cassamo U. Mussagy, Matheus S. T. Arantes, Alessandra C. Pedro, Marcos R. Mafra, Fabiane O. Farias

**Affiliations:** 1 Department of Chemical Engineering, Polytechnique Center, Federal University of Paraná, Curitiba 81531-990, PR, Brazil; juliatrentin@ufpr.br (J.T.); matheussamponi@ufpr.br (M.S.T.A.); marcos.mafra@ufpr.br (M.R.M.); 2 Escuela de Agronomía, Facultad de Ciencias Agronómicas y de los Alimentos, Pontificia Universidad Católica de Valparaíso, Quillota 2260000, Chile; cassamo.mussagy@pucv.cl; 3 Laboratório de Biotecnologia, Universidade Tecnológica Federal do Paraná (UTFPR), Curitiba 81280-340, PR, Brazil; alecristinapedro@yahoo.com.br

**Keywords:** COSMO-SAC model, thermal stability, natural food dye, functional candy

## Abstract

The growing consumer demand for natural and eco-friendly food products motivates the development and evaluation of new and natural inputs for the food industry. So, this work explores the potential of grape pomace (GP) from winemaking, a food production residue, to obtain an anthocyanin-rich, ready-to-use extract with antioxidant activity that can confer improved color-rich gummy candies. The anthocyanins’ chemical nature and the predictive COSMO-SAC model was considered for screening the best natural eutectic mixture for anthocyanin extraction. The eutectic mixtures composed of choline chloride as a hydrogen bond acceptor and acetic and citric acids as hydrogen bond donors were selected as solvents. The extraction was performed using a high-shear disperser (Ultra-Turrax^®^) at 45 °C and was stirred at 5000 rpm for 10 min. The extracts presented high total anthocyanin content (TAC), up to 60 µg equivalent of cyaniding-3-glucoside/g of dry GP, and high antioxidant activity as determined by DPPH and FRAP assays. The phenolic profile was also determined by high-performance liquid chromatography (HPLC) and the results corroborated with the antioxidant activity of the extracts. The results also demonstrate that eutectic mixtures enhance the extraction efficiency of anthocyanins and improve their stability, making them suitable for incorporation into functional food products such as gummies, acting as natural colorants.

## 1. Introduction

In recent years, growing consumer interest in natural and eco-friendly products has driven the food industry to develop innovative foods containing natural and bioactive compounds with a focus on sustainable processes [1,2,3]. In Chile, for example, the winemaking industry generates significant amounts of grape pomace (GP) (skins, seeds, stems, and disrupted cells from the grape pulp) [4], which presents an opportunity for sustainable recovery and utilization due to being a natural source of phenolic compounds, including anthocyanins with potent antioxidant properties [5,6]. Developing new food products that incorporate GP offers several benefits, such as reduced waste and positive environmental impact, added value to the food supply chain, provision of natural sources of antioxidants and other beneficial compounds to consumers, and alignment with the increasing demand for natural, sustainable, and healthy food options [7,8].

Some food applications of GP have already been investigated. For example, GP can partially replace wheat flour in enriched biscuits, improving their antioxidant activity and hydroxyl radical scavenging capabilities without changes in vitro digestion rate and good overall sensorial acceptability [9]. Furthermore, fortifying wheat bread with GP demonstrated an increase in the antioxidant capacity of the bread samples, and sensory evaluations indicated that GP fortification significantly influenced the acidity, overall flavor, astringency, and wine-like aroma of the bread while maintaining the overall acceptability [10]. As observed, GP is a well-known by-product for applications in the food industry, including the use of anthocyanins from GP as a coloring agent in fruit and soft candies, jelly candy, gummy supplements, and other products [11,12,13]. These sweet treats typically include fruit extracts, sugars, gelling agents like gelatin or pectin, and organic acids [14,15]. Thus, incorporating anthocyanins from GP into gummies could enhance their antioxidant properties and add a visually appealing color, improving the functionality of food additives.

Before anthocyanins from GP can be effectively utilized, they must first be extracted from the biomass [16,17,18]. Traditionally, this extraction process employs acidified solvents, such as hydrochloric acid or citric acid, which help solubilize and isolate the pigments from the plant material [19,20]. However, there is a growing interest in developing extraction methods that minimize the use of hazardous chemicals [3]. Ideally, these methods would utilize non-volatile, non-flammable solvents that facilitate recycling and reuse and are derived from renewable sources [21]. One promising alternative is using eutectic mixtures, which are gaining popularity in various food industries due to their environmental and safety benefits [22,23,24]. These mixtures typically consist of two or three components that form intramolecular hydrogen bonds [22]. They include hydrogen bond acceptors (HBAs) such as organic salts like choline chloride, and hydrogen bond donors (HBDs) such as alcohols and organic acids. Some formulations may also contain up to 50% (*v*/*v*) water [23]. When developing new products for human consumption, it is crucial to ensure that the components of these mixtures are approved by food safety authorities, guaranteeing their effectiveness and safety in food applications. For instance, choline chloride ([Ch]Cl), a quaternary ammonium salt used as an HBA, is widely recognized as safe for use in food products and is commonly utilized as a source of choline in dietary supplements and animal feed [25,26]. Similarly, the food industry frequently employs HBDs such as oxalic, lactic, malic, acetic, and citric acids for flavor enhancement, preservation, stabilization, and pH adjustments [27,28].

Given the beneficial properties of [Ch]Cl and the acids mentioned above, this study aims to evaluate the extraction of anthocyanins from GP using these eutectic mixtures. Organic acids are valued for their flavor enhancement, preservation capabilities, and their role in pH adjustment, making them particularly useful in the food industry. However, the low volatility of these eutectic mixtures can lead to high recycling costs in the extraction process. To address this issue, this study explores the practical application of these mixtures in the food industry, specifically investigating their use in formulating gummies enriched with eutectic mixtures rich in anthocyanin extracts. This approach highlights the potential of using food industry waste materials like GP in food formulations, with an emphasis on improving extraction protocols through the application of green solvents, not only to enhance extraction efficiency but also to align with sustainability goals in food processing. The study will evaluate several key aspects: (i) the chemistry of anthocyanins and solvent selection using an in silico solvent screening model; (ii) characterization of eutectic mixtures and GP; (iii) Ultra-Turrax-assisted solid–liquid extraction; (iv) thermal stability of the ready-to-use ingredients; (v) enrichment of gummy candies. Through this comprehensive evaluation, the research seeks to establish a sustainable method for extracting anthocyanins from GP and directly develop new products that align with sustainability and food safety principles.

## 2. Material and Methods

### 2.1. Materials

Choline chloride ([Ch]Cl) (>99.9%), acetic acid (>99.9%), citric acid (>99.9%), 2,2-diphenyl-1-picrylhydrazyl (DPPH•) (95%), and 2,4,6-Tris (2-pyridyl)-s-triazine 9 (TPTZ) were purchased from Sigma-Aldrich (St. Louis, MO, USA). All other chemicals used in this study were analytical grade and purchased from common sources. GP was obtained from *Vitis vinifera* L. plants of the Carménère variety, sourced from an organic vineyard in Valparaíso, Chile, prior to the onset of wine production during the 2023 growing season. The GP was collected, dried at 50 °C in a vacuum dryer, milled (250 µm), and stored. For gummy candy preparation, gelatin, glucose syrup, and saccharose were purchased from the local market.

### 2.2. COSMO-SAC Model

The molecular structures of interest were acquired from the PubChem Platform in SDF format https://pubchem.ncbi.nlm.nih.gov/, accessed on 1 March 2024) and then imported into the Avogadro software (https://avogadro.cc/, accessed on 1 March 2024), where molecular conformations underwent optimization to minimize activation energy. Following optimization, the molecules were loaded into the GAMESS Quantum Chemistry package to generate their sigma profile (σ-profile) [29] as described by Ferrarini et al. [30]. To gauge the solute–solvent interaction, the COSMO-SAC model was employed. This evaluation used the activity coefficient at infinite dilution (ln γ_i_∞) at 45 °C and was conducted using the JCOSMO software (version 2.9.15) developed by Gerber and Soares [23] with the GMHB1808 multi-hydrogen bond parametrization, available for free at (https://doi.org/10.5281/zenodo.3613786, accessed on 1 March 2024) [31]. A software program written in Python was developed to optimize the solvent screening.

### 2.3. Preparation and Characterization of Eutectic Mixtures

The [Ch]Cl and selected HDB were weighed at a fixed molar ratio, and the eutectic mixtures were prepared using mild heating combined with stirring at 323.15 K.

The thermogravimetric analysis was carried out in a Perkin Elmer, Waltham, MA, USA (TGA 4000) using nitrogen at a flow rate of 50 mL min^−1^. Samples were placed inside platinum pans and heated to 650 °C at a rate of 10 °C min^−1^ until complete thermal degradation was achieved.

The viscosity of the eutectic mixtures was determined experimentally using a viscometer (Brookfield DV-II+ Pro, Middleboro, MA, USA) coupled to a thermostatic bath (Vivo RT4, Seelbach, Germany) for temperature control (25–55 °C). The sample (approximately 10 mL) was placed in the sample holder, and a platinum cylinder was submerged. The analysis was started once the desired temperature was achieved and stabilized (15 min). The inner cylinder was rotated at different shear rates (100–145 s^−1^), and the shear stress was recorded at each point. The Rheocalc 1.3 software calculated the apparent viscosity, and an average apparent viscosity was determined for each temperature in the shear rate evaluated range. Finally, the Arrhenius equation (Equation (1)) was fitted to the mean apparent viscosity to determine the pre-exponential and activation energy parameters.

(1) $$\eta \left(T\right)=\eta_{o}\exp\left(-\frac{E_{a}}{R\;T}\right),$$

where *η* (mPa s) is the apparent viscosity in the temperature *T* (K), *η*_0_ (mPa s) is the pre-exponential factor, *E_a_* (J mol^−1^) is the activation energy, and *R* (8.314 J mol^−1^ K^−1^) is the universal gas constant.

### 2.4. Ultra-Turrax-Assisted Solid–Liquid Extraction

The extractions were carried out at 45 °C and at a sample/solvent ratio of 1:30. The samples were added to a glass-jacketed cell connected to a circulating bath for temperature control and submitted to a stirring (5000 rpm) in a high-shear disperser (T25, Ultra-Turrax^®^-IKA, Staufen, Germany) for 10 min.

### 2.5. Extract Characterization

The obtained extracts were characterized concerning their total anthocyanin content (TAC) [32] and total flavonoid content (TFC) [33], with some modifications [34]. The antioxidant activity of the extracts was measured by DPPH [35] and FRAP [36] assays, with some modifications as described in our previous work [34].

The identification and quantification of phenolic compounds in the samples were carried out using high-performance liquid chromatography coupled with a diode array (HPLC–DAD) (Waters, Miliford, MA, USA), Hypersil BDS C18 column (Waters, Miliford, MA, USA) (250 × 6 mm, 5 μm). Samples were filtered through a 0.22 μm *nylon* syringe filter (Millipore, São Paulo, SP, Brazil). The conditions were determined using methods 1 and 2, as shown below. The injection volume was 10 μL, and the flow rate was 1 mL/min. The analysis was performed at 35 °C, and the run time was 60 min. The quantification of compounds was carried out using calibration curves of phenolic acid and flavonoid standards.

Method 1: The mobile phase consisted of acetonitrile (A) and acidified water (0.1% acetic acid) (B). The gradient: up to 10 min 5% A; 10–50 min 95% A; from 50–55 min 5% A, maintained until 60 seg for stabilization. This method separated phenolic acids and some flavonoids.

Method 2: This method separated flavonoids; the run time was 45 min. The mobile phase consisted of acetonitrile (B), methanol (C), and acidified water (0.1% phosphoric acid). The gradient: 3% B + 2% C, 3% B + 5% C up to 5 min, 5% B + 15% C up to 10 min, 5% B + 25% C up to 25 min, 30% B + 30% C up to 30 min, and the column was stabilized for the initial conditions between 35 and 45 min.

### 2.6. Extracts’ Stability at Light and Temperature Exposure

The stability of anthocyanin-rich extracts was evaluated based on thermal degradation and light exposure. The effect of the temperature was analyzed at 25 °C, 45 °C, and 65 °C in the presence and absence of light. The total anthocyanin content retained (TAC %) was measured in intervals of 24 h through TAC analysis, and the stability was calculated by determining the reaction rate constant $\left(k\right)\;$(Equation (2)). The half-life time $\left(t_{1/2}\right)$ (Equation (3)) was calculated using a first-order reaction model for 50% TAC retention.

(2) $$\ln(C/C_{0})=-kt,$$

(3) $$t_{\left(1/2\right)}=-\frac{\ln\left(0.5\right)}{k},$$

where t is the time of storage (days), and $C_{0}$ and C are the initial and final concentrations of TAC, respectively.

### 2.7. Gummy Candy Production

For the control, gummy candy was developed in the following steps. Firstly, two solutions were prepared, as described:

- Solution 1: gelatin solution (50 wt. %) was prepared by dissolving gelatin in distilled water and stored for 30 min for gelatin hydration.
- Solution 2: a mixture of water, glucose syrup, and saccharose at a 1:1:2 mass ratio.

In sequence, solutions 1 and 2 were mixed at 1:2 (mass ratio), and the mixture was heated at 100 °C until fully dissolved. For the gummies with added extract, the fully dissolved mixture was quickly cooled until around 80 °C, and the extracts were added. Four extract concentrations were evaluated: 2.5, 5, 7.5, and 10 wt. %. After some preliminary tests, better solubilization was observed if the extracts were previously diluted in water (50 wt. %). So, the water amount for this was removed from the total amount from Solution 2, maintaining the proposed extract concentration in the final mixture. Lastly, the mixtures were transferred into starch molds and left at 25 °C for 24 h.

#### Gummy Analysis

The pH measurement was carried out according to Abinaya and co-workers [37]. The moisture was measured using an oven at 105 °C until it achieved constant weight. The water activity (a_w_) was measured using Aqualab 3TE^®^ equipment (Decagon Devices, Inc., Pullman, WA, USA).

The texture properties were evaluated using a Brookfield Texture Analyzer equipped with a cylindrical probe with a diameter of 38.1 mm. The gummy candies were compressed at a cross-head speed of 1 mm/s and a load cell capacity of 2.5 kg. Hardness, springiness, cohesiveness, gumminess, and chewiness were measured [38]. The color parameters of the gummies (L*, a*, and b*) were measured using a HunterLab^®^ Colorimeter (Reston, VA, USA).

### 2.8. Statistical Analysis

The Shapiro–Wilk (*p* ≥ 0.05) and Bartlett tests were applied to verify the hypothesis of normality and homoscedasticity of the results, respectively. The Kruskal–Wallis test was used for the non-parametric data. Analysis of variance ([ANOVA], *p* ≤ 0.05) was applied to data with confirmed normality. The Tukey test assessed the mean differences without a control sample, and Fischer’s least significant difference (LSD Fisher) tested assays with the control sample. The results were expressed as a mean ± standard deviation (*n* = 3). All statistical analyses were performed using the software Statistica^®^ 10.0.

## 3. Results and Discussion

### 3.1. Anthocyanin Chemistry and Solvent Selection

Concerning the wide range of solvents available for use as extractant agents for anthocyanin recovery and our aim to save time and resources, it was carried out in silico solvent screening. For this, the main anthocyanins found in the grape pomace and the chemical nature of these compounds were considered.

Chemically, anthocyanins, a flavonoid subclass, are glycosides of anthocyanidins, their chemical species pH-dependent. Structurally, they are based on a flavylium nucleus bonded with sugar, hydroxyl groups, and organic acids [39,40]. In the pH range commonly found in plants and foods (2 < pH < 8), it can present different chemical characteristics, e.g., charge and electronic distribution, resulting in different colors and modulation of its reactivity and interactions in the vegetal/food matrix and its components [40]. At acidic conditions, the flavylium cation (AH^+^) is the predominant species, which is also the most stable form, responsible for the reddish/pink color of the anthocyanins.

**Figure 1:**
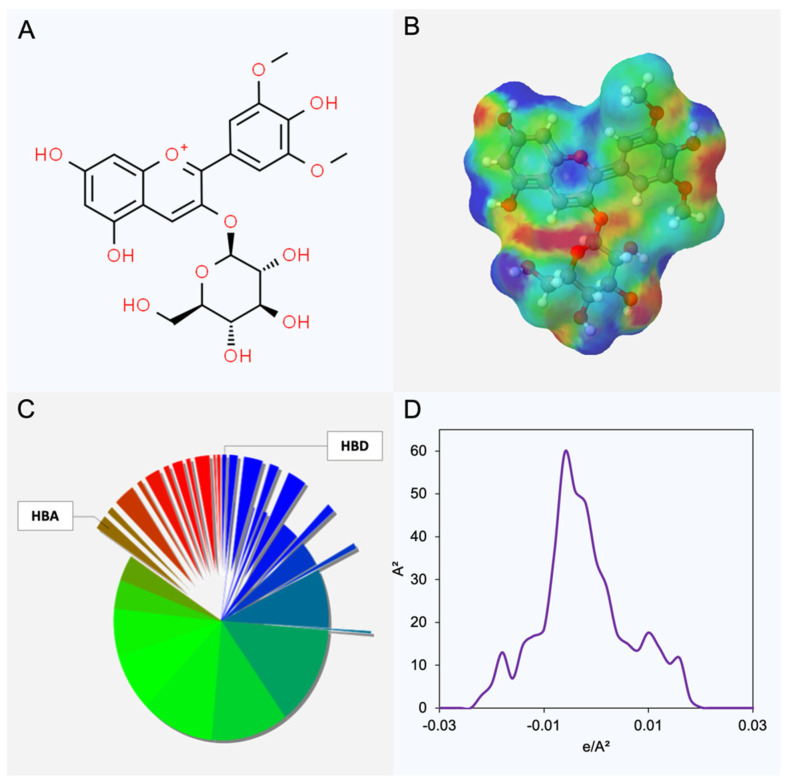
Chemical structure (**A**), charge distribution (**B**,**C**), and σ-profile of the malvidin-3-glucoside (**D**). Note: In Figures (**C**,**D**), red and blue colors are related to the HBA and HBD regions of the molecule, respectively. The green color is related to the neutral area.

In the grape pomace, the anthocyanins found by HPLC–DAD analyses were malvidin-3-glucoside, malvidin-3–acetoglucoside, delphinidin-3-glucoside, cyanidin-3-glucoside, petunidin-3-glucoside, and peonidin-3-glucoside [41], which were considered as target molecules for the solvent screening. In addition, considering the superior stability of anthocyanins’ color in an acid medium, all the molecules were positively charged. As an example, Figure 1 shows the chemical structure, charge distribution, and sigma profile (σ-profile) of the malvidin-3-glucoside.

The color differences in Figure 1B,C result from the charge distribution around the molecule and respective induced charges. The red and blue regions correspond to the positive and negative induced charges, respectively. The green regions, in turn, are the neutral charges.

The red and blue regions can also be described as the hydrogen bond acceptor (HBA) and hydrogen bond donor (HBD) regions of the respective molecule, which are measured in Figure 1D. Figure 1D corresponds to the pie chart of the distribution charge, where it is possible to show that around 17 and 27% of the malvidin-3-glucoside molecule can act as HBA and HBD, respectively. Finally, Figure 1D presents the *σ*-profile of the malvidin-3-glucoside. The *σ*-profile is a graphical representation of the surface charges, where the *x*-axis (e/A^2^) is the charge density, and the *y*-axis (A^2^) is the respective area of the molecule where the density is shown [42]. The malvidin-3-glucoside *σ*-profile presents a significant neutral area (between 0.01 and −0.01 e/A^2^), as well as polar regions (0.01 < e/A^2^ < −0.01).

The properties of the malvidin-3-glucoside and other anthocyanins were considered for the solvent choice, and the screening was carried out using eutectic mixtures based on organic acids as solvents. This class of compounds was chosen considering reports in the literature of eutectic mixtures composed of organic acids, as HBD presented a significantly higher capacity to dissolute and extract anthocyanins than other classes of HBD [43,44]. This can be attributed to the low pH values of the acid-based DES, which improve its ability to solubilize polar and nonpolar compounds due to the high capacity to donate protons and accept electrons [45].

**Figure 2:**
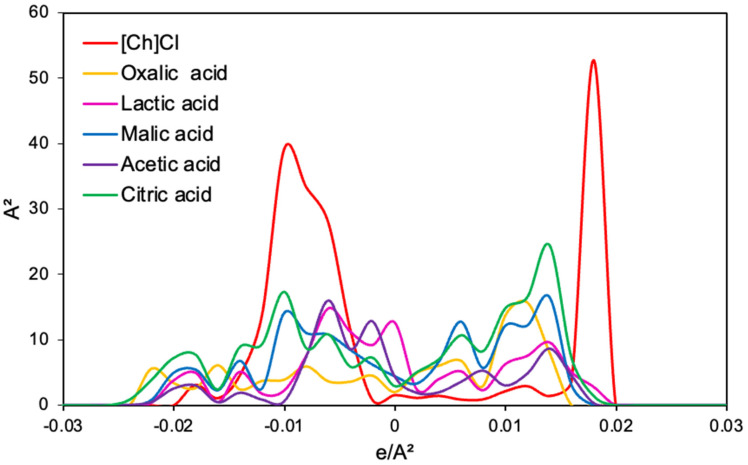
σ-profile of eutectic mixtures precursor: choline chloride ([Ch]Cl, HBA) and HBD (oxalic, lactic, malic, acetic, and citric acids).

Figure 2 shows the σ-profile of the HBA (choline chloride) and HBD (oxalic, lactic, malic, acetic, and citric acids) under evaluation as solvents for the recovery of anthocyanins. The polar regions of the eutectic mixture precursors are complementary to those of the malvidin-3-glucoside (Figure 1D), corroborating the ability of these solvents to solubilize anthocyanins.

**Figure 3:**
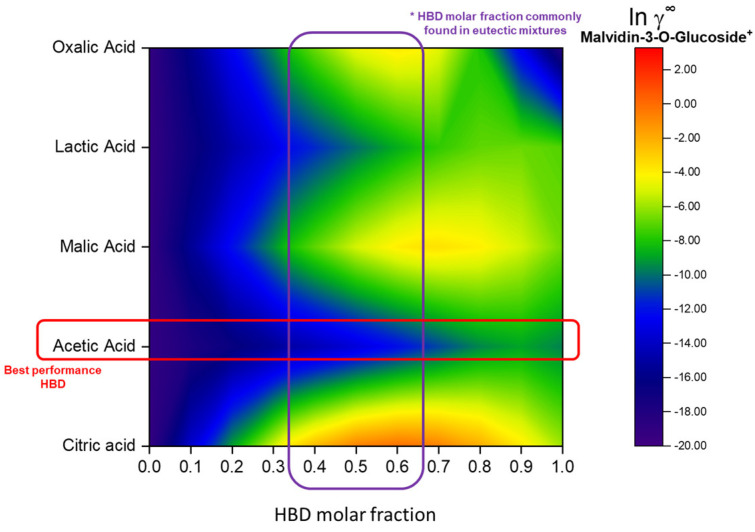
HBD screening for [Ch]Cl-based eutectic mixtures aiming for anthocyanin extraction.

Finally, to select the best HBD and the HBA/HBD molar ratio, the potential of each mixture to solubilize the anthocyanins was evaluated through the coefficients in infinite dilution (ln ɣ^∞^) for anthocyanins in each eutectic mixture, as shown in Figure 3 (for delphinidin-3-glucoside, cyanidin-3-glucoside, petunidin-3-glucoside, and peonidin-3-glucoside, cf. Supplementary Material). Lower values of ln ɣ^∞^ are related to higher solute–solvent affinity, i.e., higher capacity of the solvent to solubilize the target compound. So, the eutectic mixture of [Ch]Cl and acetic acid (AA) is a potential solvent. Considering the HBA/HBD molar ratio usually practiced for eutectic mixtures, the mixture [Ch]Cl: AA at a 2:1 molar ratio was selected for the following steps.

In addition, considering the potential application in candy gummies as *ready-to-use* extracts, the mixture composed of citric acid (CA) as HBD ([Ch]CL: CA, 2:1) was also taken into consideration. This mixture was selected due to the well-established use of CA as a food additive.

In silico tools, such as the COSMO-SAC model, have already been successfully applied for green solvent screening and a better comprehension of solute–solvent interactions [46,47,48,49]. The main advantages of this approach are related to the wide range of compounds able to be used in green solvents obtained, saving time and resources avoiding extensive “*trial and error*” laboratory tests. Also, it is important to highlight that these predictive models cannot consider the solvents’ physical properties, such as density and viscosity, which directly influence mass transfer processes [50]. The high viscosity is one of the major disadvantages of eutectic mixtures concerning the extraction processes’ requirements.

### 3.2. Eutectic Mixtures Characterization

Firstly, the selected eutectic mixtures were properly prepared. However, both present a considerably high viscosity that does not favor the mass transfer for extraction purposes. So, we add 30 wt. % of water in the selected eutectic mixtures. In this step, the in silico screening by the COSMO-SAC model was carried out again for all solvents, and the affinity behavior was the same as that presented in Figure 3 (in the absence of water). Also, no significant changes in the ln ɣ^∞^ behavior were observed—cf. Supplementary Material.

The water addition in organic-acid-based eutectic mixtures was also reported in the literature. According to Benvenutti and co-workers [45], the water presence does not affect the good capacity of the eutectic mixtures to solubilize the anthocyanin, besides contributing to the viscosity reduction and reducing the costs of the solvent. Guo and co-workers evaluated the effect of water (0 to 50 wt. %) addition in eutectic solvents composed of [Ch]Cl: CA: Glucose (1:1:1) for anthocyanin recovery, and the extraction yield reached a maximum of 30 wt. % of water [51]. The eutectic mixtures, added to 30 wt. % of water, were characterized concerning their thermal stability by TGA and viscosity measurement.

**Figure 4:**
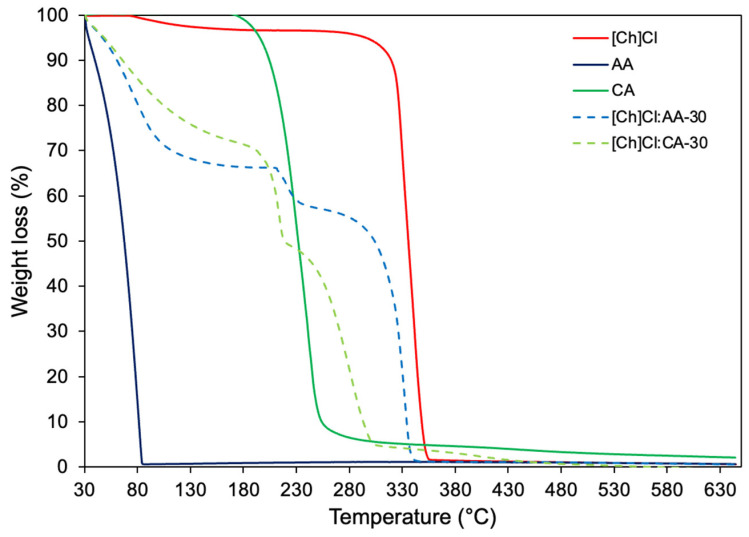
Thermogravimetric curves of [Ch]Cl, acetic acid, citric acid, [Ch]Cl: AA-30, and [Ch]Cl: CA-30.

The TGA analysis was carried out to measure the thermal stability of the eutectic mixtures. For process purposes, thermal stability is essential in knowing the temperatures where materials do not suffer degradation or significant damage to their physical–chemical properties. Figure 4 shows the thermal degradation of the acetic acid, citric acid, choline chloride, and respective eutectic mixtures under evaluation. At 84 °C, the AA is completely degraded, while CA and [Ch]Cl degradation initiate at 180 and 300 °C, respectively. For the eutectic mixtures, two events are seen; the first one, around 100 °C, is related to the water added to the mixtures. The second one, for both of them, initiates around 230~240 °C until complete degradation of the solvent.

Viscosity is related to a fluid’s resistance in response to deformation, so this parameter is essential for operations where agitation or pumping of fluids is necessary. The eutectic mixtures’ viscosity was measured from 25 to 55 °C, and atmospheric pressure (91 kPa), as shown in Table 1. As expected, solvents’ viscosity decreases with temperature due to the decrease in H-bonding interactions in the eutectic mixture with the rising temperature.

The Arrhenius model adjusted the experimental data to obtain the activation energy (E_a_). The E_a_ can be described as the energy barrier of a fluid to shear stress; so, higher values of E_a_ are related to strong interactions within the fluid and more difficulty moving. Higher values of E_a_, on the other hand, indicate a higher dependency of viscosity with the temperature: the same variation at the temperature (the increase from 25 to 45 °C, for instance) promotes a significatively higher decrease in the viscosity of the eutectic mixture composed of CA when compared to the mixture composed of AA.

The eutectic mixture of AC, as HBD, was the most viscous. It can be attributed to the AC structure, with three carboxyl groups, responsible for stronger molecular interactions with the HBA than AA, which has only one carboxyl group. For comparison, at 40 °C, pure water has a viscosity of around 0.67 mPa·s [52], significantly lower than both mixtures for all temperatures under evaluation. These findings, combined with the molecular contribution evaluated by the COSMO-SAC model, give us important information concerning the solute–solvent affinity and involved transport phenomena and the solvation and extraction ability of each solvent.

### 3.3. Ultra-Turrax-Assisted Solid–Liquid Extraction

Envisioning a fast and effective method to extract anthocyanins using eutectic mixtures, the Ultra-Turrax-assisted extraction was applied in this work. The Ultra-Turrax promotes a high-sheer dispersion and is indicated to achieve short extraction times, besides improving the mass transfer in high-viscosity solvents [53].

**Figure 5:**
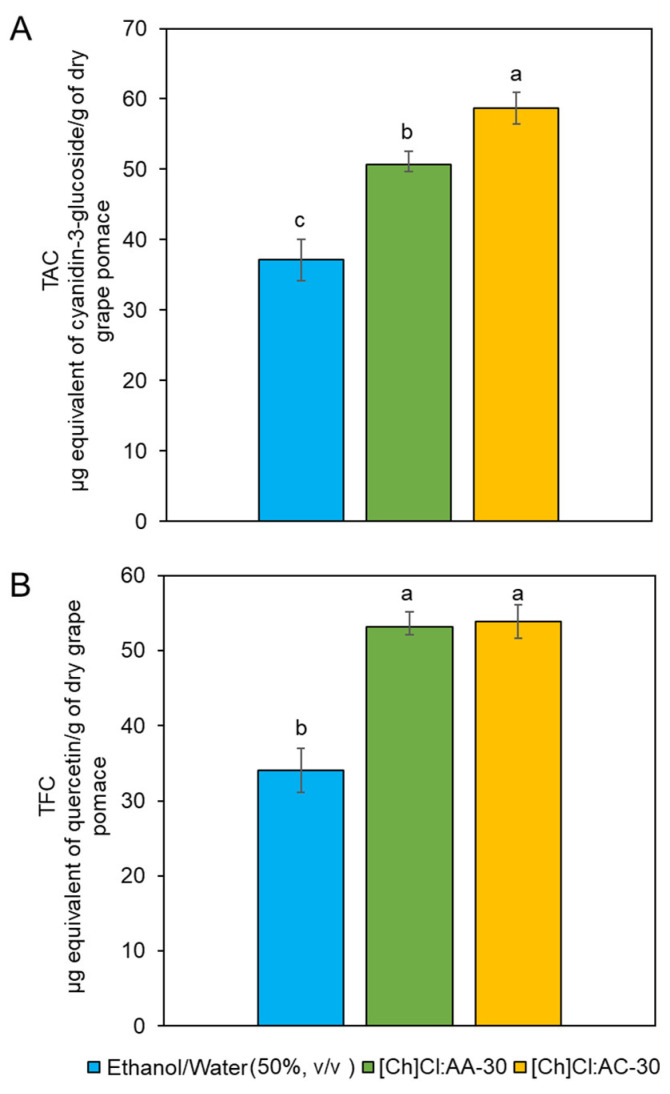
Extraction yields for the three solvents under evaluation. The extraction conditions were 45 °C, a solid/solvent ratio of 1:30, 10 min, and Ultra-Turrax stirring of 5000 rpm. (**A**) Total anthocyanin content (TAC) µg equivalent of cyanidin-3-glucoside/g of dry grape pomace. (**B**) Total flavonoid content (TFC) µg equivalent of quercetin/g of dry grape pomace. Note: Different letters represent significant differences according to the Tukey test (*p* < 0.05).

The temperature extraction of 45 °C was fixed considering the significant viscosity reduction, mainly for [Ch]Cl: CA-30. The select temperature contributes to the mass transfer phenomena but is not too high to cause anthocyanin degradation [51]. The extraction yields in terms of TAC are shown in Figure 5. For comparison, the extraction was also carried out using a hydroalcoholic solvent (ethanol/water, 50% *v*/*v*), which allows us to confirm the great ability of eutectic mixtures to extract anthocyanins. In addition, the Ultra-Turrax was demonstrated to be effective in achieving a high extraction index in a short process time.

The obtained extracts were also characterized concerning their antioxidant activity through DPPH and FRAP analysis (Table 2) and profile of phenolic compounds by HPLC (Table 3).

As shown in Table 3, the main phenolic acids identified in the samples were gallic and syringic acid, while the main flavonoids were cyanidin, isoquercetin, and quercetin. Furthermore, these compounds showed significant differences between the solvents used, demonstrating that the molecular structure and the presence of multiple compounds in the extracts can alter the adsorption behavior in each solvent. These factors are fundamental for predicting biological health effects and food applications [54].

Studies in the literature show that these compounds have important biological activities in the human body, being promising for use as functional ingredients. Recent studies show that gallic acid has anti-hyperglycemic activity, determined by increasing β-cell activity and glucose uptake by peripheral tissue, causing increased insulin sensitivity [55]. Syringic acid is scarcely reported in the literature, but studies emphasize its inhibitory effect and its dependence on concentration and time by studying cell proliferation in hormone-sensitive breast cancer lines. Furthermore, this phenolic acid can prevent obesogenic diet-induced weight gain and insulin resistance [56].

Flavonoids have been related to preventing the inflammatory response caused by oxidative stress, inhibiting the levels of interleukin (IL)-1β, IL-6 and tumor necrosis factor (TNF)-α, and restoring IL-10 levels. Prevention of the inflammatory response suggests a reduction in the risk of developing cardiovascular and neurological diseases [57]. Studies suggest that cyanidin can counteract BPA-induced oxidative stress, improve hippocampal and cortical neurogenesis, and interact with Wnt signaling proteins [58]. Kim et al. [59] showed that quercetin and isoquercetin extracted from *Elaeocarpus sylvestris* inhibited the replication of varicella-zoster virus (VZV) and human cytomegalovirus (HCMV) in vitro. Isoquercetin has also been linked to the prevention of neurochemical and neurobehavioral changes in vivo [60].

As observed in our study (Table 3), syringic acid and isoquercetin presented the highest concentrations per liter of sample compared to the other identified compounds. Subiría-Cueto et al. [54] also found isoquercetin to be the most abundant phenolic compound in grape pomace (*Vitis vinifera*). Furthermore, the isoquercetin and other individual compounds were best fitted to the Freundlich isotherm model, indicating multilayer binding. Thus, this isotherm model may suggest that the different concentrations of isoquercetin and other compounds obtained (Table 3) have a strong relationship with the form of interaction between these substances and the solvents tested.

The study carried out by Fontana et al. [56] also showed that syringic acid (720–6665 µg/g) was the most abundant phenolic acid in the pomace of grapes grown in Argentina. Still, in the same study, the authors correlated the levels of the main anthocyanin present in the samples, malvidin-3-glucoside, with the levels of syringic acid. Thus, samples with higher levels of malvidin-3-glucoside showed higher levels of syringic acid, an effect also observed in our study. Positively correlating the content of total anthocyanins (Figure 5), cyanidin (Table 3), and syringic acid (Table 3), our samples presented a decreasing order of concentration: [Ch]Cl: AA-30 > [Ch]Cl: CA-30 > Ethanol/water.

Furthermore, studies have shown that anthocyanins and syringic acid can act synergistically and contribute to the antioxidant activity of grape pomace [56]. In the study carried out by Milinčić et al. [61], hydroxybenzoic acids, including syringic acid, were the most important compounds for the scavenging activity of ABTS+ and DPPH radicals. Our study also observed this relationship, demonstrating the synergism between syringic acid and anthocyanins present in the samples.

Thus, the results show that the phenolic compounds identified make an important contribution to the antioxidant activity of the samples. Therefore, the extracts obtained can potentially be used to develop products with functional purposes.

### 3.4. Thermal Stability

**Figure 6:**
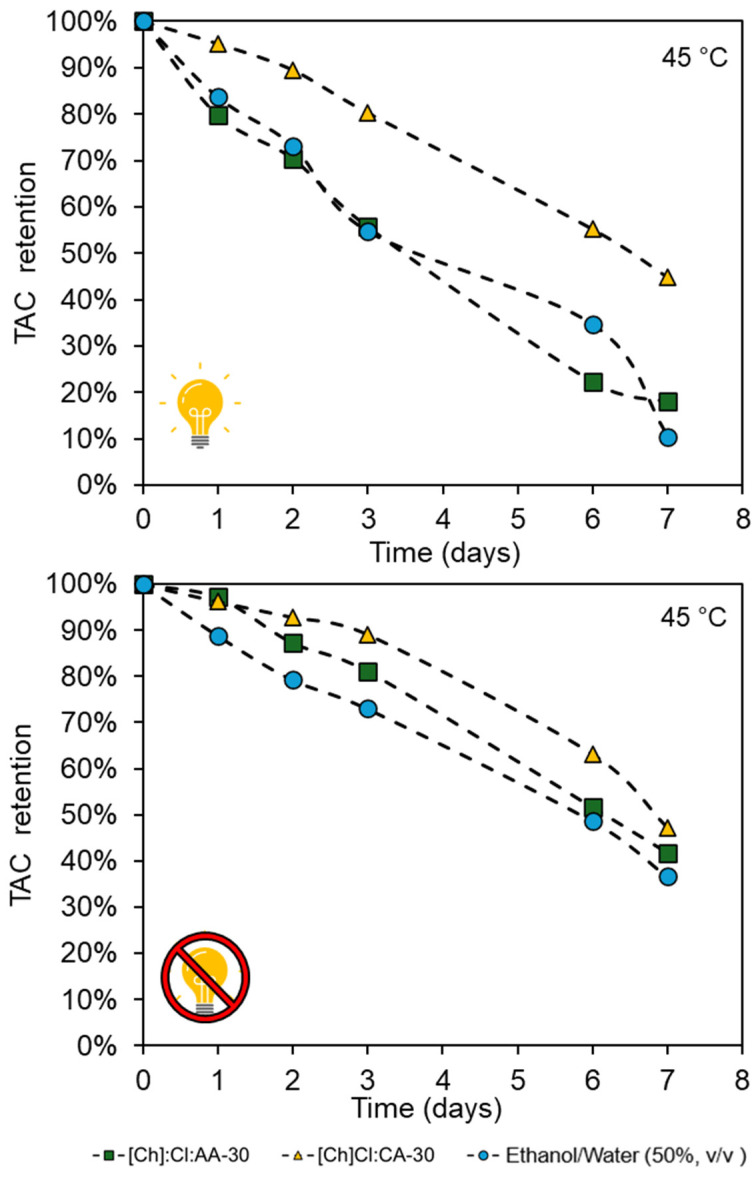
Total anthocyanin content (TAC) retention after the exposure of the extracts to light and dark conditions and a temperature of 45 °C.

To determine if the eutectic mixtures can promote a protective effect on the anthocyanins, thermal stability in the presence and absence of light was evaluated at 25, 45, and 65 °C. In Figure 6, it is possible to see the kinetics curves for all solvents at 45 °C (for 25 and 65 °C, cf. Supplementary Material).

The kinetics curves calculated the half-life time (t_1/2_) for each solvent and condition, as shown in Table 4. As can be seen, both light and temperature negatively affect anthocyanin stability, with TAC retention lower in lighted conditions and at high temperatures. In general, TAC preservation was greater in the eutectic mixtures than in the hydroalcoholic solvent, which can be related to the physical–chemical properties of these solvents.

Furthermore, it is important to highlight the superior preservative effect of [Ch]Cl: CA-30, which can be due to two factors, including hydrogen bonds between the solvent and the anthocyanins and the high viscosity of this solvent, which can contribute to reducing the solute mobility, i.e., the contact of anthocyanins with oxygen is reduced [38].

### 3.5. Gummy Candy Development

**Figure 7:**
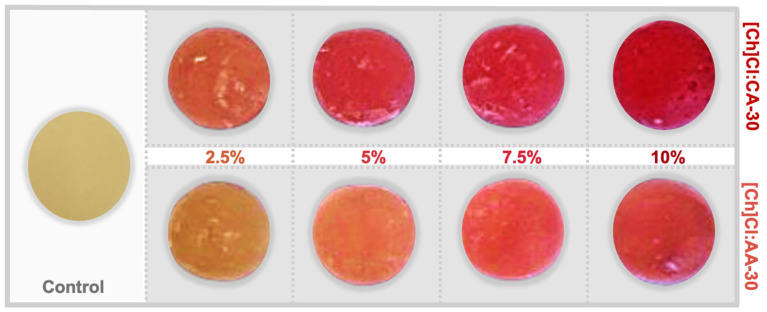
Pictures of gummy candies: control sample and added anthocyanin-rich extracts at different concentrations.

Eutectic mixtures of [Ch]Cl and organic acids are potential solvents for ready-to-use extracts. This work evaluated them as natural colorants for the development of gummy candies. These extracts can act as natural colorants, besides contributing to this product’s antioxidant activity. The final appearance of the gummy candies, with different extract concentrations, is shown in Figure 7.

In Figure 6, it is also possible to see a significant visual improvement of the pink color with the increase of the extract concentration in the gummies. So, the color differences were properly measured using a colorimeter; the results are shown in Table 5. The L* values correspond to the brightness of the samples (black to white), which was reduced with the increase of extracts in the gummies. The a* values correspond to color changes from green (negative values) to red (positive value). As expected, the a* parameter increases with the extract concentration due to the pink/reddish color conferred by the anthocyanins. Finally, the b* values correspond to the color changes from blue (negative values) to yellow (positive values), so the lower values of b* were expected with the increase of extract concentration.

The moisture content and water activity were also measured (*cf.* Table 4). The moisture content ranged from 23.7% for the control sample to 30.2 g/100 g. The increase of the moisture content with the increase of the concentration of the extract is a result of the processes produced. Considering the high temperatures achieved during gummy production, the extracts were mixed with water (1:1 mass fraction) to avoid anthocyanin degradation, and added to the gummy at the end of the process. Despite the water amount to solubilize the extract being removed from the total water added for gummy production, the high moisture can result from the addition when the heat conditions are not as rigorous.

The water activity (a_w_) of the gummies ranged from 0.822 to 0.849, which is in agreement with the expectations for gummy candies (0.6 to 0.9) [40]; this is an important parameter due to its effect on the quality, shelf-life, and sensory of the candies.

The effect of the extract concentration on the texture properties was also measured, as shown in Table 6, considering the impact of these properties on consumer acceptance. The hardness is the maximum force applied in the first compression to deform the sample, so less energy is required for the deformation with the increase of extract concentration. The increase of extract amount on gummy development results in a higher heterogeneity of the network structure, contributing to lower hardness values [41]. The springiness is related to the height that some food recovers during the time between the first and second bite, so higher values of springiness result in the mastication energy in the mouth [42]. Cohesiveness was the parameter less affected by extract addition. Gumminess and chewiness are related to the energy for semisolid food disintegration and the energy required to masticate a solid food to a state ready for swallowing, respectively. Both parameters were lower with the increase in extract concentration and are related to the primary parameters of hardness, springiness, and cohesiveness.

Finally, a visual analysis of the first signals of deterioration (fungal growth) was carried out on the gummy candies that were properly packed and stored at room temperature (around 25 °C). For the control gummy and those with 2.5 wt. % of [Ch]Cl: AA-based extract added, the fungal growth was observed after 20 days of storage, while those with 10 wt. % of the same extract added presented a longer shelf-life, with fungal growth observed only after 40 days of storage. When the eutectic mixtures were composed of acetic acid, as HBD, no deterioration signals were observed after 60 days of storage for all extract concentrations. No significant visual changes in color were observed during the storage time.

## 4. Conclusions

This work successfully developed a method for extracting anthocyanins from grape pomace (GP) using natural eutectic mixtures, which resulted in a total anthocyanin content (TAC) of up to 60 µg equivalent of cyanidin-3-glucoside/g of dry GP. The extraction was optimized using an Ultra-Turrax-assisted solid–liquid extraction at 45 °C and 5000 rpm. This led to significant antioxidant activity, with DPPH radical scavenging reaching 85% and robust reducing power indicated by FRAP assays. The phenolic profile, characterized by HPLC, confirmed the presence of several beneficial compounds correlating with the observed antioxidant activity. Furthermore, incorporating these anthocyanin-rich extracts into gummy candies demonstrated promising colorimetric and sensory qualities, showcasing the potential of GP as a sustainable source of natural colorants and health-promoting ingredients in the food industry. This research highlights the valorization of winemaking by-products and aligns with the growing consumer demand for natural and eco-friendly food products.

## Figures and Tables

**Table 1:** Eutectic mixtures viscosity at different temperatures and Arrhenius model adjusted.

Temperature (°C)	Viscosity (mPa·s)	
	[Ch]Cl: AA-30	[Ch]Cl: CA-30
25	13.01	22.46
35	11.85	16.21
45	11.33	13.27
55	10.47	11.96
Arrhenius model adjusted		
ln(η_0_)	0.27	−3.84
E_a_ (kJ/mol)	5.67	17.12
R^2^	0.99	0.96

**Table 2:** Antioxidant activity in anthocyanin-rich extracts.

Sample	Antioxidant Activity µg Equivalent of Trolox/g of Dry Grape Pomace	
	DPPH	FRAP
[Ch]Cl: AA-30	462.24 ± 23.32 ^b^	384.62 ± 15.29 ^a^
[Ch]Cl: CA-30	515.72 ± 11.00 ^a^	129.45 ± 3.45 ^c^
Ethanol/water	474.71 ± 13.52 ^b^	212.69 ± 8.55 ^b^

Note: Different letters in the same column represent significant differences according to the Tukey test (*p* < 0.05).

**Table 3:** Profile of phenolic compounds by HPLC.

Sample	Concentration (mg/L)				
	Gallic Acid	Syringic Acid	Cyanidin	Isoquercetin	Quercetin
[Ch]Cl: AA-30	1.043 ± 0.004	4.516 ± 0.005	2.268 ± 0.296	4.808 ± 0.184	1.545 ± 0.055
[Ch]Cl: CA-30	0.988 ± 0.004	4.252 ± 0.053	0.999 ± 0.050	6.406 ± 0.079	0.999 ± 0.031
Ethanol/water	1.171 ± 0.013	3.693 ± 0.053	0.891 ± 0.064	9.146 ± 1.379	1.907 ± 0.053

**Table 4:** Half-life time (t_1/2_) of total anthocyanin retention after exposure to light and temperature.

Solvent	t_1/2_ (Days) Light			t_1/2_ (Days) Dark		
	25 °C	45 °C	65 °C	25 °C	45 °C	65 °C
[Ch]Cl: AA-30	10.57	2.90	0.87	29.88	6.23	1.21
[Ch]Cl: CA-30	25.67	6.78	0.99	57.28	8.03	1.31
Ethanol/water	6.22	2.77	1.31	28.41	5.34	1.94

**Table 5:** Characterization of the gummy candies with added anthocyanin-rich extracts.

Sample	Color Analysis			Water Activity (a_w_)	Moisture Content (g/100 g)	pH
	L*	a*	b*			
Control	43.42 ± 2.81 ^a^	−0.17 ± 0.01 ^f^	15.30 ± 1.65 ^a^	0.822 ± 0.007 ^e^	23.706 ± 0.506 ^e^	3.86 ± 0.04 ^c^
CA-2.5%	34.74 ± 1.01 ^b^	7.57 ± 0.60 ^d^	10.95 ± 0.85 ^b^	0.826 ± 0.001 ^d,e^	26.423 ± 0.421 ^d^	3.45 ± 0.06 ^d^
CA-5%	29.58 ± 1.19 ^c^	13.76 ± 0.67 ^b^	7.71 ± 0.45 ^c^	0.834 ± 0.001 ^b,c^	28.396 ± 0.584 ^c^	3.39 ± 0.04 ^d^
CA-7.5%	23.22 ± 0.95 ^d,e^	15.21 ± 0.16 ^a^	3.90 ± 0.14 ^d,e^	0.835 ± 0.001 ^b,c^	29.763 ± 0.222 ^b^	3.19 ± 0.01 ^e^
CA-10%	14.10 ± 2.37 ^f^	10.52 ± 1.38 ^c^	1.43 ± 0.77 ^f^	0.833 ± 0.004 ^b,c^	29.812 ± 0.260 ^b^	3.10 ± 0.01 ^f^
AA-2.5%	32.57 ± 3.43 ^b,c^	3.92 ± 0.77 ^e^	11.30 ± 2.13 ^b^	0.832 ± 0.001 ^c,d^	26.404 ± 0.146 ^d^	4.39 ± 0.03 ^a^
AA-5%	29.08 ± 2.04 ^c^	7.28 ± 0.50 ^d^	8.69 ± 1.11 ^c^	0.840 ± 0.001 ^b^	28.207 ± 0.231 ^c^	4.14 ± 0.01 ^b^
AA-7.5%	24.18 ± 0.90 ^d^	7.69 ± 1.03 ^d^	5.47 ± 0.84 ^d^	0.848 ± 0.001 ^a^	29.096 ± 0.918 ^b,c^	4.09 ± 0.01 ^c^
AA-10%	20.05 ± 1.91 ^e^	7.60 ± 0.96 ^d^	2.78 ± 0.59 ^e,f^	0.849 ± 0.004 ^a^	30.187 ± 0.171 ^a^	3.00 ± 0.01 ^g^

Note: Different letters in the same column represent significant differences according to the Fischer LSD test (*p* < 0.05).

**Table 6:** Texture properties of anthocyanin-enriched gummy candies.

Sample	Hardness (N)	Springiness (mm)	Cohesiveness	Gumminess (N)	Chewiness (J)
Control	74.09 ± 7.98 ^a^	4.15 ± 0.22 ^a^	0.78 ±0.07 ^e, f^	54.99 ± 2.42 ^a^	0.28 ± 0.11 ^a^
CA-2.5%	42.19 ± 6.97 ^c^	4.17 ± 0.05 ^a^	0.76 ± 0.02 ^f^	28.25 ± 6.96 ^b^	0.13 ± 0.03 ^b^
CA-5%	17.14 ± 1.39 ^d^	4.22 ± 0.17 ^a^	0.81 ± 0.03 ^d, e, f^	13.92 ± 1.18 ^d^	0.06 ± 0.00 ^b, c^
CA-7.5%	14.17 ± 1.08 ^d^	4.58 ± 0.56 ^a^	0.78 ± 0.03 ^e, f^	9.89 ± 2.30 ^c^	0.04 ± 0.01 ^c^
CA-10%	7.47 ± 0.54 ^e^	4.79 ± 0.95 ^a^	0.82 ± 0.01 ^c, d, e^	6.11 ± 0.40 ^e^	0.03 ± 0.01 ^c^
AA-2.5%	13.93 ± 1.14 ^d^	4.41 ± 0.76 ^a^	0.90 ± 0.03 ^a^	12.57 ± 0.71 ^d^	0.05 ± 0.01 ^b, c^
AA-5%	16.35 ± 2.35 ^d^	4.29 ± 0.32 ^a^	0.87 ± 0.01 ^a, b, c^	14.22 ± 1.89 ^d^	0.06 ± 0.01 ^b, c^
AA-7.5%	7.71 ± 1.82 ^e^	4.68 ± 0.70 ^a^	0.87 ± 0.02 ^a, b^	6.74 ± 1.48 ^e^	0.03 ± 0.01 ^c^
AA-10%	4.77 ± 0.72 ^f^	4.46 ± 0.65 ^a^	0.85 ± 0.02 ^c, d, e^	47.92 ± 3.59 ^b^	0.22 ± 0.03 ^a^

Note: Different letters in the same column represent significant differences according to the Fischer LSD test (*p* < 0.05).

## Data Availability

The original contributions presented in the study are included in the article/Supplementary Material, further inquiries can be directed to the corresponding author.

## Supplementary Materials

The following supporting information can be downloaded at: https://www.mdpi.com/article/10.3390/foods13172840/s1, Figure S1: HBD screening for [Ch]Cl-based eutectic mixtures aiming for anthocyanins extraction; Figure S2: HBD screening for [Ch]Cl-based eutectic mixtures with 30 wt. % of water aiming for anthocyanins extraction; Figure S3: Total anthocyanin content (TAC) retention after the exposure of the extracts at light and dark conditions at 25 and 65 °C.
