# Supplementary material for: Antioxidant Ready-to-Use Grape Pomace Extracts Recovered with Natural Eutectic Mixtures for Formulation of Color-Rich Gummies
Source: Foods. 2024 Sep 7;13(17):2840. doi: 10.3390/foods13172840 (PMC11395118; doi:10.3390/foods13172840)
Supplement: Supplementary file 1 [file foods-13-02840-s001.zip › foods-3181338-supplementary.pdf]

Supplementary Material

Antioxidant Ready-to-Use Grape Pomace Extracts Recovered with Natural Eutectic Mixtures for Formulation of Color-Rich Gummies

Julia Trentin [1], Cassamo U. Mussagy [2], Matheus S. T. Arantes [1], Alessandra C. Pedro [3], Marcos R. Mafra [1] and Fabiane O. Farias [1,*]

[1] Department of Chemical Engineering, Polytechnique Center, Federal University of Paraná, Curitiba 81531-990, PR, Brazil

[2] Escuela de Agronomía, Facultad de Ciencias Agronómicas y de los Alimentos, Pontificia Universidad Católica de Valparaíso, Quillota 2260000, Chile

[3] Laboratório de Biotecnologia, Universidade Tecnológica Federal do Paraná (UTFPR), Curitiba 81280-340, PR, Brazil

* Correspondence: fabianefarias@ufpr.br

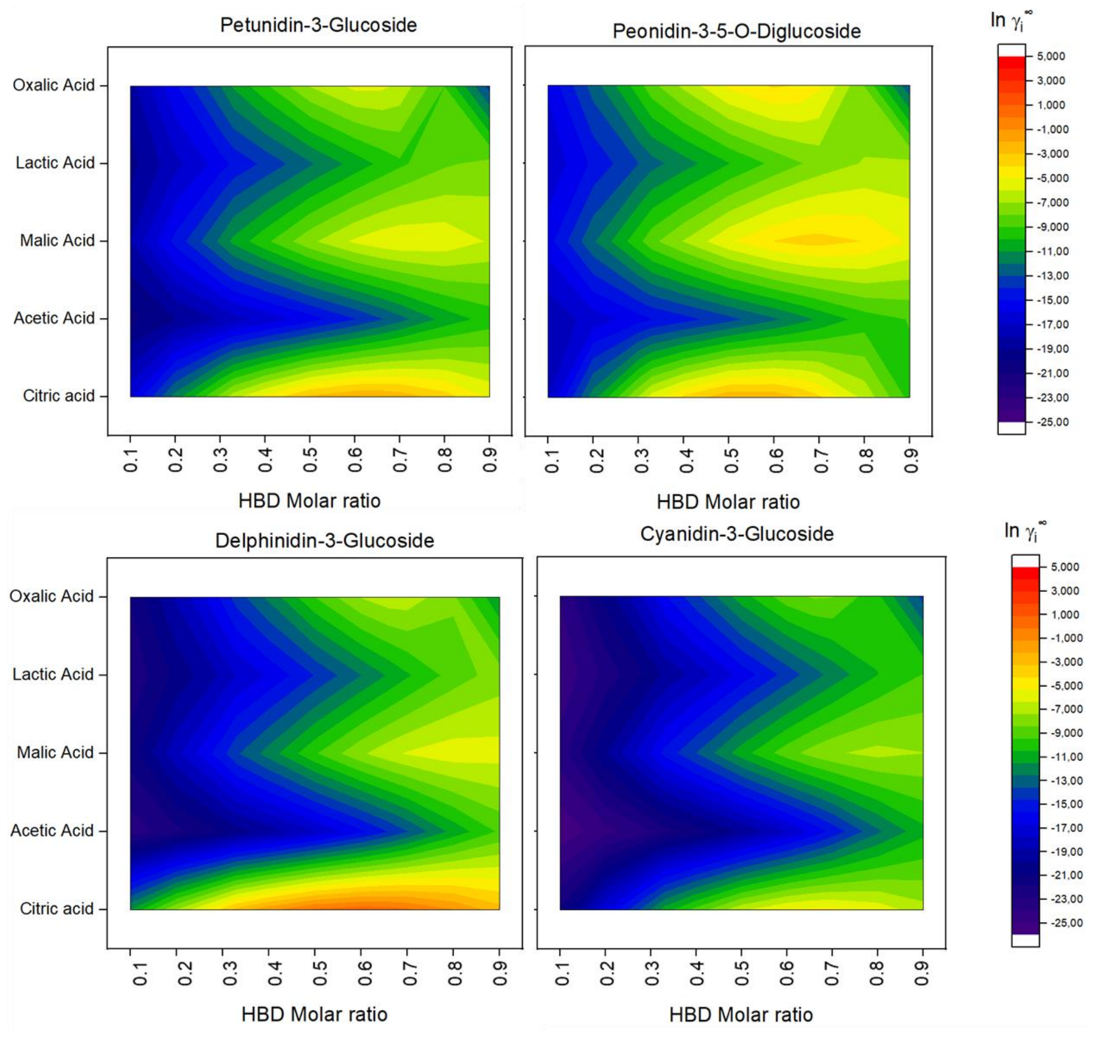

Figure S1. HBD screening for [Ch]Cl-based eutectic mixtures aiming for anthocyanins extraction

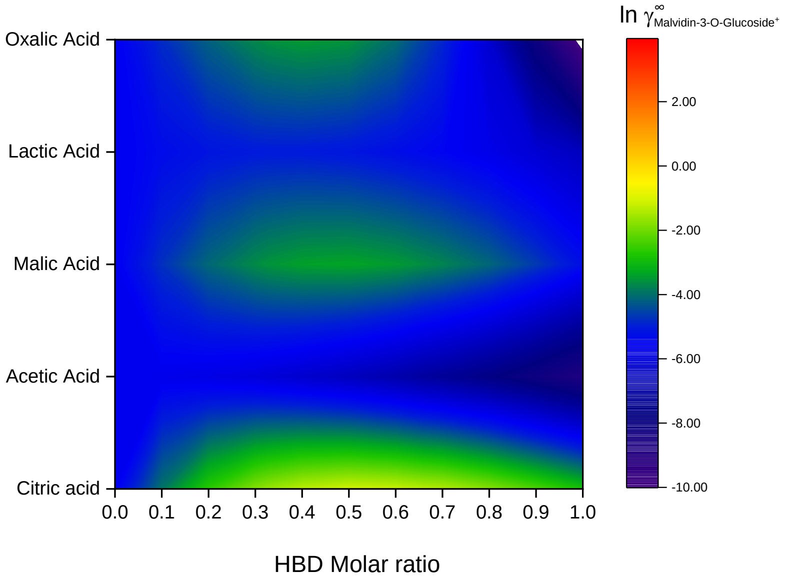

Figure S2. HBD screening for [Ch]Cl-based eutectic mixtures with 30 wt.% of water aiming for anthocyanins extraction

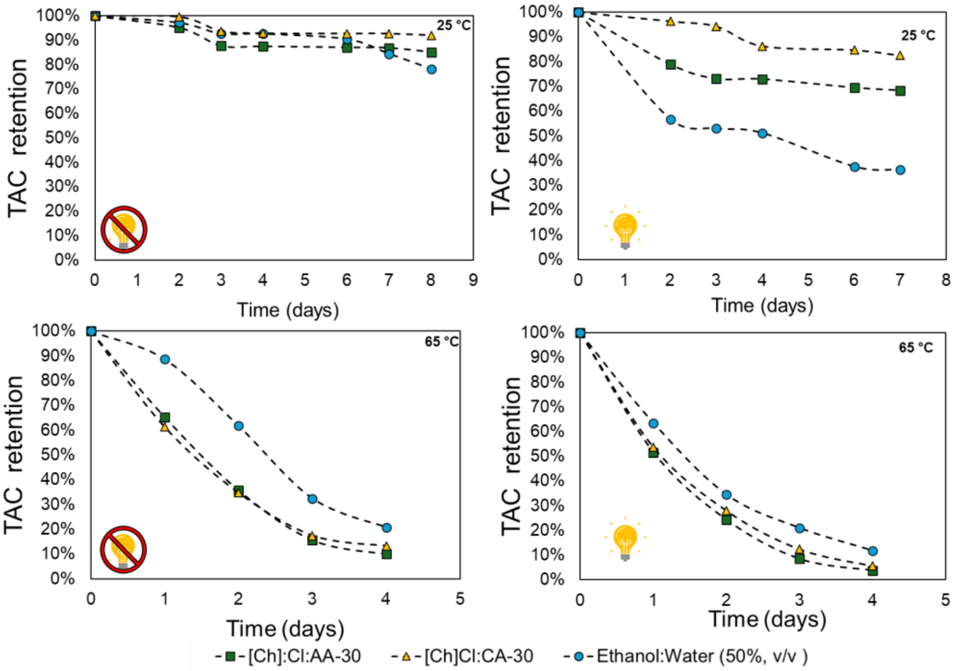

Figure S3. Total anthocyanin content (TAC) retention after the exposure of the extracts at light and dark conditions at 25 and 65 °C.
